# Clinical characteristics of acute pancreatitis in pregnancy: experience based on 121 cases

**DOI:** 10.1007/s00404-017-4558-7

**Published:** 2017-11-21

**Authors:** Lingyu Luo, Hao Zen, Hongrong Xu, Yin Zhu, Pi Liu, Liang Xia, Wenhua He, Nonghua Lv

**Affiliations:** Department of Gastroenterology, The First Affiliated Hospital of Nanchang University, No.17 Yongwai Zheng Street, Nanchang, 330006 China

**Keywords:** Acute pancreatitis in pregnancy, Clinical characterization, Maternal and fetal mortality

## Abstract

**Purpose:**

Acute pancreatitis in pregnancy (APIP) is a rare condition; however, it markedly affects maternal and fetal health. This study aimed to describe the types, clinical characteristics, mortality, and the safety and necessity of gestation termination of acute pancreatitis in pregnancy (APIP).

**Methods:**

We retrospectively reviewed 121 APIP cases in the Gastroenterology Department of The First Affiliated Hospital of Nanchang University. APIP diagnosis were based on 2012 Atlanta Criteria. The correlation between APIP types, severity, biochemical parameters and mortality was analyzed.

**Results:**

The most common symptoms for APIP were abdominal pain (86.8%) and vomiting (73.6%). The most common causes for APIP were gallstone (36.4%) and hypertriglyceridemia (32.2%) and hypertriglyceridemic APIP was correlated with a higher rate for local complication (*P* = 0.012). Serum calcium level was negatively correlated with the severity of APIP (*P* < 0.01). The overall maternal and fetal mortality rate were 3.3% (4/121) and 11.6% (14/121), respectively. The severity of APIP was significantly correlated with higher risks for maternal and fetal death (*P* < 0.01). 72.7% of moderate-to-severe APIP patients underwent Cesarean section to terminate gestation safely.

**Conclusion:**

The most common causes of APIP were gallstone and hypertriglyceridemia. Lower level of serum calcium could be used as an indicator for the severity of the APIP. The severity of APIP was associated with higher risk for neonate asphyxia, and maternal and fetal death.

## Introduction

Acute pancreatitis in pregnancy (APIP) is a rare condition with an approximate incidence of 1 over 1000–12,000 pregnant women [1–4]. The acute onset and difficulty in diagnosis and treatment of APIP significantly threaten the maternal and fetal health [5, 6]. It was previously demonstrated that the mortality rate was approximately 37% for the mother and 60% for the fetus, whereas more recently the numbers have decreased significantly due to the improvements in the diagnosis technique, intensive and neonatal care [2, 7–9].

To date, most paper summarizing the clinical characteristics of APIP were of small sample size. A number of factors have been acknowledged as the pathogenic cause for APIP, among which gallstone remains to be mostly common [10]. Other risk factors were also proposed such as increased maternal age, increased pregnancy number, high fat diet as well as higher body mass index [11]. The diagnosis of APIP is often complicated by other obstetrical emergencies. Therefore, it is crucial for understanding the clinical characteristics of APIP. However, as to the diagnosis and treatment, the specific guideline of APIP is still absent, the major reason probably is related to low incidence rate and scarce clinical data.

In this study, we retrospectively reviewed 121 cases of APIP from 2005 to 2015 in our center to describe the types, clinical characteristics, mortality, and the safety and necessity of gestation termination of acute pancreatitis in pregnancy (APIP) i.

## Materials and methods

### Patients

This study recruited pancreatitis patients from the previously established pancreatitis database from 2005. Pregnant patients from September 2005 to July 2015 were included in this study. Inclusion criteria were acute pancreatitis diagnosed during pregnancy.

### APIP diagnosis and definition

The acute pancreatitis in pregnancy diagnosis was fulfilled based on Atlanta criteria [12] when at least two of the following: (1) acute upper abdominal pain radiated to the back; (2) serum amylase or lipase level was three times higher than normal; (3) radiological evidence indicates acute pancreatitis. APIP was also divided into three severity categories. Mild acute pancreatitis (MAP) referred to pancreatitis without organ dysfunction or generalized complications. Moderate to severe pancreatitis (MSAP) referred to pancreatitis with transient organ dysfunction or localized/generalized complication within 48 h after treatment. Severe pancreatitis (SAP) referred to pancreatitis with persistent organ dysfunction or localized/generalized complication for more than 48 h after treatment. Organ dysfunction was defined based on the modified Marshall scoring [12]. The localized complication referred to acute peri-pancreatic fluid collection (AFPC), pancreatic pseudocyst, acute necrosis and encapsulated necrosis [12]. The generalized complication referred to two of the following: body temperature > 38 °C or < 36 °C; white blood cell count > 12,000/mm^3^ or < 4000/mm^3^; heart rate > 90 bpm, respiratory rate > 20/min or PCO_2_ < 32 mmHg.

APIP was also categorized by different pathogenic causes. Acute gallstone pancreatitis was diagnosed by an increased ALT level > 150 U/L within 48 h of onset, as well as radiological evidence of abdominal ultrasonography and magnetic resonance cholangiopancreatography (MRCP) [13]. Hypertriglyceridemic pancreatitis (HTGP) was diagnosed based on Chinese guidelines for the management of acute pancreatitis (Shanghai, 2013) [14] with either a serum triglyceride ≥ 11.3 mmol/L or serum triglyceride was between 5.65 and 11.3 mmol/L with a lipid turbidity appearance after excluding gallstone, alcohol or medication factors. Idiopathic pancreatitis was diagnosed with radiological evidence of pancreatitis after excluding gallstone, alcohol, hypertriglyceridemia, medication, trauma, autoimmune and surgical factors [13].

### Termination of pregnancy

Pregnancy was determined to be terminated by experienced obstetricians and gastroenterologists. The indications were confirmed fetal death in utero, obligation to use fetal-toxic medication for pancreatitis or organ failure. All termination was consent by the patient after comprehensive evaluation of surgical risks. The approaches for pregnancy termination included Cesarean section, natural birth including preterm and termed birth, and natural or drug induced abortion.

### Laboratory examination and definition

Complete blood count was examined by automatic analyzer (SySMex XN2000, Sysmex Corporation, China). Blood glucose, lipids, and electrolytes were performed by an automatic biochemical analyzer (Hitachi 7600, Hitachi Limited, Japan). Serum fasting glucose was examined by glucose oxidase method. Serum electrolytes including potassium, sodium and chloride were examined by the indirect ion selective electrode method and calcium was tested by Arsenazo III method. Serum triglyceride was examined by the glycerol phosphate oxidase peroxidase method. Hyperglycemia was defined as a fasting glucose ≥ 7.8 mmol/L and hypertriglyceridemia was defined as a fasting serum triglyceride ≥ 11.3 mmol/L. Hypocalcaemia was defined as serum calcium < 1.75 mmol/L and an increased white blood cell count was defined as a white blood cells count > 1 × 10^10^/L.

### Statistical analysis

All data were analyzed by SPSS 17.0 (SPSS Statistics, Chicago, IL, USA). Quantitative data were compared by *t* test, whereas categorical data were compared by χ^2^ Chi square test. Data were compared by non-parametrical test if it was not distributed normally. *P* value < 0.05 was considered statistically significant.

## Results

### APIP patient characteristics

**Fig. 1:**
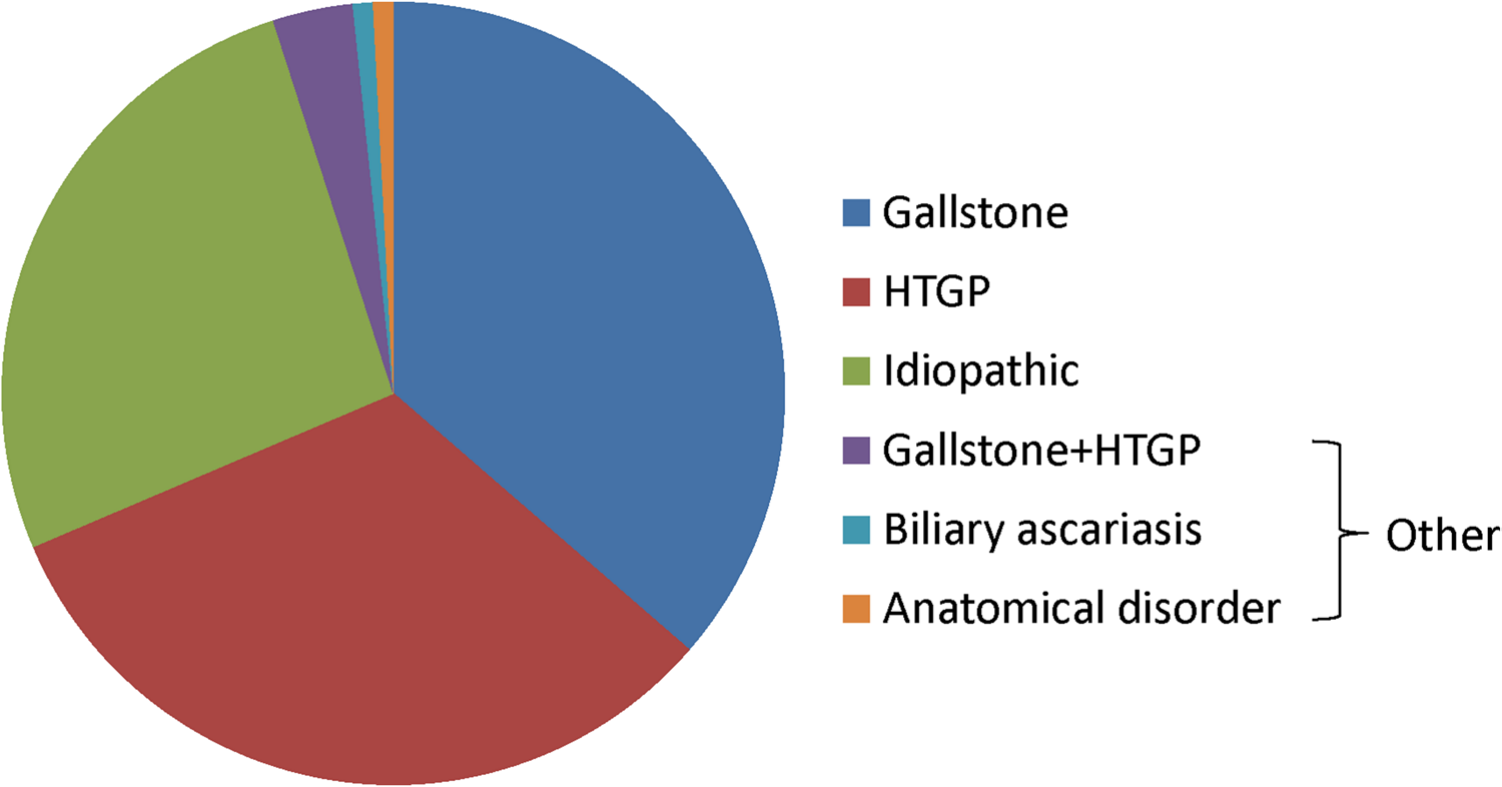
Patient proportion by different causes of APIP. HTGP: hypertriglyceridemic pancreatitis

Patient characteristics are presented in Table 1 and the pathogenic causes for APIP are shown in Fig. 1. The average age of our patients was 27.8 ± 5.4 years. Most patients were at gestational week 24–40 at the time of APIP diagnosis (67.8%, 82/121), whereas we noted that 37 patients had APIP during the second trimester and 2 patients during the first trimester. The main causes for APIP were gallstone (36.4%), hypertriglyceridemia (32.2%) and idiopathic pancreatitis (26.4%) (Fig. 1). Other causes of APIP included biliary ascariasis (1 case), anatomical disorder (1 case) and gallstone complicated with hypertriglyceridemia (4 cases) (Fig. 1). 49% (59) were mild acute pancreatitis (MAP), 36% (44) moderate to severe acute pancreatitis (MSAP) and 15% (18) were severe acute pancreatitis (SAP).

**Table 1:** APIP patients characteristics by different pathogenic types

	Gallstone	HTGP	Idiopathic	*P* value
Number	44	39	32	
Age*	28 ± 5.3	28.7 ± 5.4	26.8 ± 5.8	0.331
Gravidity*	2.0 ± 1.0	2.3 ± 1.3	2.5 ± 2.0	0.85
Parity*	1 ± 1	0.9 ± 0.9	1.0 ± 1.1	0.16
Trimester of pregnancy on admission, *n* (%)				
Early (1–12 weeks)	1 (2.3)	0 (0)	1 (3.1)	0.88
Mid (12–24 weeks)	16 (36.4)	15 (38.5)	11 (34.4)	
Late (24–40 weeks)	27 (61.4)	24 (61.5)	20 (62.5)	
Gestational weeks on admission	29.7 ± 6.9	28.6 ± 7.7	33.7 ± 4.5	0.06
Interval between diagnosis and delivery	1.8 ± 1.3	1.8 ± 1.3	2.3 ± 1.8	0.09
Severity of APIP*, *n* (%)				
MAP	24 (54.5)	12 (30.8)	18 (56.3)	0.091
MSAP	15 (34.1)	18 (46.2)	12 (37.5)	
SAP	5 (11.4)	9 (23.1)	2 (6.2)	
Localized complications, *n* (%)				
APFC	13 (29.5)	20 (51.3)	6 (18.8)	0.012
Pancreatic pseudocyst	1 (2.3)	3 (7.7)	1 (3.1)	
Acute necrosis	1 (2.3)	3 (7.7)	1 (3.1)	
Encapsulated necrosis	1 (2.3)	3 (7.7)	0 (0)	
Organ dysfunction, *n* (%)				
Respiratory	13 (29.5)	14 (35.9)	9 (28.1)	0.742
Circulation	1 (2.3)	2 (5.1)	1 (3.1)	
Kidney	2 (4.6)	4 (10.3)	1 (3.1)	
Multiple organ dysfunction	3 (6.8)	4 (10.3)	1 (3.1)	
History of hypertension, *n* (%)	0 (0)	1 (2.6)	1 (3.1)	
History of diabetes, *n* (%)	0 (0)	6 (15.4)	1 (3.1)	
Fatty liver disease, *n* (%)	5 (11.4)	7 (17.9)	3 (9.4)	0.518
Ascite, *n* (%)	17 (38.6)	20 (51.3)	9 (28.1)	0.137
Pleural effusion, *n* (%)	20 (45.5)	27 (69.2)	17 (53.1)	0.131

Quantitative data were presented as number and percentage and compared by χ^2^ Chi square test

*APIP* acute pancreatitis in pregnancy, *HTGP* hypertriglyceridemic pancreatitis, *MAP* mild acute pancreatitis, *MSAP* moderate to severe acute pancreatitis, *SAP* severe acute pancreatitis, *APFC* acute peri-pancreatic fluid collection

*Data were compared using non-parametrical test

We further compared clinical characteristics among three major pathogenic causes. The severity of APIP was not significantly different among causes of APIP (*P* = 0.09) (Table 1). When comparing the pancreatitis complications, the hypertriglyceridemia APIP had the highest proportion of acute peri-pancreatic fluid collection (*P* = 0.012). There is no difference between other pancreatitis complications, gestational characteristics, and organ dysfunction or diseases history among different causes (Table 1).

### Clinical manifestations of APIP

In this study, we found that abdominal pain and vomit remained the two most predominant clinical symptoms in our patients. The location of abdominal pain was mainly in the upper abdomen, which occurred in 86.8% patients (105/121) whereas only 11.6% had lower (5.0%, 6/121) or generalized abdominal pain (6.6%, 8/121). More than half of the patients had vomit (73.6%, 89/121) and fever was less common (23.1%, 28/121). The major finding after physical examination included abdominal tenderness (81.8%, 99/121) and rebound tenderness (34.7%, 42/121). The location of abdominal tenderness was mainly at the upper abdomen (91%, 90/99), whereas only 3% occurred at the lower abdomen (3/99) and 6% at the whole abdomen (6/99).

### Laboratory abnormality and severity of APIP

Some commonly used laboratory results were compared among MAP, MASP and SAP based on the severity (Table 2). Only the level of serum calcium was negatively correlated with the severity of APIP (*P* < 0.01). However, serum glucose, triglycerides, or white blood cell count was not correlated with the severity of APIP (Table 2).

**Table 2:** Severity of APIP and abnormality of serum examination

	MAP	MSAP	SAP	*P* value
Number	59	44	18	
Hyperglycemia	10 (16.9)	13 (29.5)	7 (38.9)	0.111
Hypertriglyceridemia	10 (16.9)	13 (29.5)	7 (38.9)	0.185
hypocalcaemia	3 (5.1)	7 (15.9)	8 (44.4)	< 0.01
Increased white blood cell count	45 (76.3)	36 (81.8)	15 (83.3)	0.712

Data were presented as number (percentage) and compared by Chi square test; hyperglycemia was defined as fasting glucose ≥ 7.8 mmol/L; hypocalcaemia was defined as serum calcium < 1.75 mmol/L; hypertriglyceridemia was defined as serum triglyceride ≥ 11.3 mmol/L; increased white blood cell count was defined as white blood cells > 1 × 10^10^/L

*MAP* mild acute pancreatitis, *MSAP* moderate to severe acute pancreatitis, *SAP* severe acute pancreatitis

### Maternal and fetal outcomes

#### Maternal and fetal mortality

In our study, there were 4 cases of maternal death (with fetal death) in total (3.3%). One was due to cardiac sudden death (HTGP, at week 13) and 3 were due to multiple organ dysfunctions (1 HTGP, at week 37; the other 2 cases were biliary pancreatitis, at week 38 and 13, respectively). Besides the 4 cases of maternal with fetal death, another 10 cases of fetal death occurred (Table 3), among which 5.1% of fetal death occurred in the MAP group, 6.8% in MASP, and 44.4% in the SAP group. The mortality rate was positively correlated with the severity of APIP (*P* < 0.01). However, it was not significantly different among different pathogenic causes of APIP (*P* = 0.08, Table 4).

**Table 3:** Severity, pathogenic types of APIP and fetal mortality

	Living	Dead	*P* value
Number	107	14	
Severity of APIP, *n* (%)			
MAP	56 (52.3)	3 (21.4)	< 0.01
MSAP	41 (38.3)	3 (21.4)	
SAP	10 (9.3)	8 (57.1)	
Pathogenesis of APIP, *n* (%)			
Gallstone	39 (36.4)	5 (35.7)	0.08
HTGP	31 (29.0)	8 (57.1)	
Idiopathic	31 (29.0)	1 (7.1)	

Data were presented as number (percentage) and compared by Chi-square test

*APIP* acute pancreatitis in pregnancy, *MAP* mild acute pancreatitis, *MSAP* moderate to severe acute pancreatitis, *SAP* severe acute pancreatitis, *HTGP* hypertriglyceridemic pancreatitis

**Table 4:** Apgar score of living neonates delivered by Cesarean birth (*N* = 48)

	Apgar score			*P* value
	0–3	4–7	8–10	
MAP	0	3	14	< 0.01
MSAP	1	10	15	
SAP	2	1	2	

Data were presented as number by each category

*MAP* mild acute pancreatitis, *MSAP* moderate to severe acute pancreatitis, *SAP* severe acute pancreatitis

Due to pancreatitis Apgar scores at 5 min of the neonates were evaluated in 48 live Cesarean births (Table 4). It was demonstrated that asphyxia in the neonates was significantly correlated with the severity of APIP of the mother (*P* < 0.01).

The maternal and fetal mortality is recorded in Table 5. Among 121 patients, Gestation was terminated by different approaches, mainly based on fetal and maternal conditions.

**Table 5:** Maternal and fetal mortality by different APIP severity

	MAP	MSAP	SAP
Total number	59	44	18
Total life birth, *n* (%)	55	40	10
Continued pregnancy	35	8	3
Live Cesarean birth	18 (30.5)	32 (72.7)	6 (33.3)
Termed birth	2 (3.4)	0	1 (5.6)
Total dead birth, *n* (%)	4	4	8
Dead Cesarean birth	0	2 (4.5)	2 (11.1)
Preterm birth	1 (1.7)	1 (2.3)	0
Drug induction labor	2 (3.4)	1 (2.3)	2 (11.1)
Natural abortion	1 (1.7)	0	0
Maternal and fetal death	0	0	4 (22.2)

*APIP* acute pancreatitis in pregnancy, *MAP* mild acute pancreatitis, *MSAP* moderate to severe acute pancreatitis, *SAP* severe acute pancreatitis

## Discussion

This case series study described 121 cases of acute pancreatitis in pregnancy in Chinese and showed that the severity of the APIP was correlated with risk for neonate asphyxia, as well as maternal and fetal death. To our knowledge, this is so far the largest study focusing on acute pancreatitis in pregnancy.

Our data confirmed that the most common causes for acute pancreatitis in pregnancy were gallstone and hypertriglyceridemia in Chinese. Alcohol was considered rare in Chinese maternal population due to a relatively lower rate for Chinese women who regularly takes alcohol drinks [15]. Idiopathic APIP also accounted for 26% in our population. Although we have ruled out other possible causes before the diagnosis of idiopathic APIP, it remained possible that some of the idiopathic APIP was partially due to high triglyceride level which had dropped after a few days of fasting. Our data also demonstrated that half of the HTGP had acute peri-pancreatic fluid collection. Similarly, previous studies also pointed out that HTGP is more threatening than other types of APIP [16, 17]. These data indicated that HTGP was associated with higher risk for localized complications.

In our study, most patients had APIP onset during the third trimester (68%) but we also had 2 and 31% had APIP during the first and the second trimester. This proportion was similar from previous studies reporting that most APIP occurred in the third trimester [18, 19]. The underlying mechanism is probably due to the compression to the pancreas and gallbladder by the enlarging uterus, as well as changing in steroids that directly affect gallbladder function [20]. Bolukbas et al. also found that even in early pregnancy, a significant drop in gallbladder ejection fraction was demonstrated [21]. Our data also showed the negative relations between serum calcium and the severity of APIP, which is consistent in non-pregnant pancreatitis patients. A testing of serum calcium may indicate the severity of the disease and more studies are warranted to confirm this.

Our results also demonstrated a close relationship between severity of APIP and maternal and fetal outcome. In our study, once there was proof of single or multiple organ failures for the patient, we consulted the obstetric doctors to assess the safety and feasibility of the operation for terminating pregnancy to rescue the lives of APIP patients and the fetus. It could reduce the effect of those facilitating factors due to gestation itself as well as save the fetus as much as possible. A total of 4 maternal deaths (3.3%) occurred all in the SAP group. SAP group also had the highest rate for fetal death (44%). In severe pancreatitis, systematic inflammation cytokine explosion could lead to generalized endothelial injury, which cause further tissue damage. A previous study by Sun et al. also indicated that the increased intra-abdominal pressure during SAP was also associated with higher risk of fetal death [22]. On the other hand, it was also noted that 42.9% of fetal death occurred in the MSP and MASP groups. One possible reason was that severe APIP during early pregnancy is particularly threatening to fetal life due to the severe onset of the disease [23]. Taken together, the severity of APIP determines the risk for neonatal health, and maternal and fetal death.

Termination of pregnancy might be also a key and necessary step for the treatment of APIP. Our data showed that the interval between diagnosis of APIP and delivery were 2.1 ± 1.3, 1.8 ± 1.4 and 2.9 ± 2.3 days for MAP,MSAP and SAP patients, respectively. For the patients with APIP, organ dysfunction will be assessed on admission at 48 h after hospitalization, and the termination of pregnancy will be suggested if the following situations exist: (1) when single or multiple organ dysfunction exists, continuing pregnancy might aggravate the disease; (2) during early or midtrimester of pregnancy, clinical medications might affect the growth of the fetus; (3) fetal development is basically mature (after 37 weeks), SIRS caused by AP and clinical medications might increase the risk of fetal death. Moreover, we found that MSAP patients underwent the highest rate (72.7%) of Cesarean section to get live birth. The reasons might be: (1) most of APIP was occurred in the third trimester and the development of the fetus was relatively mature; (2) the general condition of MASP patients was usually stable enough to complete the operation; (3) Most MASP patients preferred to terminate the pregnancy beforehand to avoid potential harms to the fetus. Fortunately, no maternal death was caused by the operation. The benefits for the patients include preventing the deterioration of APIP and allowing more effective therapy without considering the status of the fetus. So once one or more indications were met, Cesarean section would be a safe and necessary option to stop the deterioration of pancreatitis and save both the mother and the baby.

In conclusion, acute pancreatitis in pregnancy is a rare but severe disease potentially threatening the maternal and fetal life. The most common causes of APIP were gallstone and hypertriglyceridemia, and APIP due to hypertriglyceridemia tends to be associated with more complications. Lower level of serum calcium could be used as an indicator for APIP severity. The severity of the APIP was correlated with higher risk for neonate asphyxia, and maternal and fetal death. Appropriate timing for termination of pregnancy is necessary and safe for APIP patients. More studies are warranted for further elucidation of the etiology, risk factors and treatments of acute pancreatitis in pregnancy, especially the severe type.
